# To the Physicians of the Western States

**Published:** 1828-07

**Authors:** Daniel Drake

**Affiliations:** Cincinnati, Ohio


TO THE

### PHYSICIANS

OF THE

### WESTERN STATES.

At the close of the last number of The Western Journal of the Medical and Physical Sciences, the subscribers were informed that it has been transferred to the undersigned, who has, therefore, unexpectedly become at once Proprietor, Editor and Publisher.

It is not without regret, that he finds himself thus situated; as his editorial duties were, of themselves, sufficient to occupy most of the time, which professional engagements left at his disposal. Inured, however, to hard labour, and deeply interested, as he has become, in the success of the undertaking, he is far from cherishing a feeling of discouragement. Having, in 1819, issued proposals for a similar work, and obtained a considerable number of subscribers, he was the first to project such an enterprize in the Western States—although various causes, chiefly growing out of his connexion with Medical Schools, precluded him from entering upon its performance before the opening of the year 1827. Since that time he has encountered several unexpected difficulties; but indulges the hope that they have at length disappeared, and that the work will be rendered as permanent, as such publications generally are: whether it can be made as worthy of public patronage, must depend, not on his own exertions merely, but on the contribution to its pages, with which he may be favored, by the enlightened Physicians, Surgeons and Naturalists of the great region which it is designed to represent.

Such contributions he, again, respectfully, solicits, and would here take occasion to indicate certain subjects, to which he would, especially, invite the attention of his Western brethren.

1. Rare or singular cases of disease. To report all cases would be impracticable and absurd. To report the successful *only*, is uncandid. Cases which go to the suggestion of a new principle or rule of practice; which either weaken or sustain a disputed point; which, terminating unfavorably, may serve as a warning to the profession; and, finally, those which occur with exceeding rarity, may always be published with advantage to others, and credit to the reporter.

2. New forms of disease, depending on causes, either discovered or unknown.

3. Annual epidemics, in which the reporter should always omit, as far as possible, whatever is common to the malady in all its visitations, and occupy himself with its peculiarities.

4. Brief or synoptic reports, consisting of a sort of account current with the weather and diseases, of the different seasons.

5. Accounts of new medicines; with a clear discrimination between their effects and the effects of those for which they are offered, either as auxiliaries or substitutes.

6. Experimental treatises on our *native* medicinal plants.

7. Chemical and therapeutick accounts of our mineral springs.

8. Comparative histories of the diseases of the negroes and whites.

9. Facts for an estimate of the peculiarities, which the diseases prevailing on board the steam boats of the Western and Southern waters may present.

10. Reports on the diseases in our penitentiaries.

11. Detached facts, in clinical medicine, which may be proper for insertion under the head of Original, Miscellaneous Intelligence.

12. Notices of the discovery in the West and South, of such minerals, as are in any way employed in pharmacy.

The Editor would not, however, absolutely restrict his correspondents to new facts; inasmuch as he believes that on many subjects a new arrangement of old ones, is imperiously required; he would be happy, therefore, to receive and publish essays and dissertations on all subjects in the profession, provided they develope, either new principles or new practical maxims.

In regard to the rules which should be observed in the preparation of papers, for a Medical Journal, he will take the liberty of offering to his junior brethren a few remarks.

In such papers, the first excellence is *truth*. Every thing that is published in a journal of medical science, should, as far as possible, be divested of error; for it may all, sooner or later, influence the treatment of the sick, whose lives might be jeopardized, in consequence of perversions or suppressions of the truth. The next great requisite is *perspicuity;* that what the writer has observed and thought, may be readily and clearly comprehended. The next is *conciseness*; which many readers would, in reality, place at the head of the column of good qualities:— And it cannot be denied, that they would have much reason on their side; for the quantity that is published, in the journals, and detached works, of the present age, is really enough to deter the timid and the indolent, from all reading. *Originality*, is another important property, which should never be wanting. Mere learning, and laborious rumination of that which has been masticated by others, will neither advance the interests of the profession, nor satisfy readers of good sense. Finally, those who write for medical journals, should not consider the minor rules of composition beneath their attention. If advanced in years and reputation, their example will be authoritative, and should, therefore, be a good one: If young, they should seize every occasion to improve themselves in the art of writing. Indeed no member of the profession, either high or low, should consider himself at liberty to disregard the laws of *orthography*, *etymology*, *syntax* and *collocation* in any thing which he presumes to address to his brethren. Lastly, in mercy to editors and compositors, he should write (or procure to be written) an unaffected and legible hand; without which, he has, in fact, no guaranty, that the printed will be a correct copy of his written communication.

The undersigned, regarding the "labourer as worthy of his hire," proposes to his brethren of the West and South:

I. A receipt for the year's subscription to the Journal, for every paper, of not less than four pages, which shall be deemed worthy of insertion.

II. One dollar a page, for every communication, exceeding four pages, which may, from its merits, be entitled to publication.

III. A premium of fifty dollars, for the best dissertation on each of the following topics:

1. The general principles of the pathology and treatment of the diseases, of the negroes of the Southern States; with a particular application of them to the malady generally denominated Negro Consumption; reference being constantly had to the constitutions and diseases of the whites of the same region, as standards of comparison.

2. The treatment of autumnal fever on the principles of Bronssais; with a comparative estimate of the success of that plan and the one previously pursued, illustrated by original cases.

3. The remote cause and morbid anatomy of the disease denominated in the Western country the "*Sick Stomach*;" with a successful discussion of the question, whether it is a new disorder.

4. The successful application of the process of Civiale, to the destruction of the *calculus vesicæ*, established by American cases.

The dissertations to be accompanied, as is usual in such cases, by private letters containing the names of the authors; which letters will not be opened until a decision is made on the respective merits of their papers.

It only remains for the undersigned to add, that, having divested himself of all connection with medical schools, he proposes to bestow upon the Western Journal, now the only publication of the kind in Cincinnati, all the time and attention that can be spared from professional duties; and that, in dedicating his humble abilities to its establishment and dissemination, he hopes to be honored with the countenance and co-operation of many of his senior brethren of the West; while he confidently anticipates the assistance of his former alumni, both private and public, wherever this appeal may find them.

DANIEL DRAKE, M. D.

Cincinnati, Ohio, August, 1, 1828.

